# Retinopathy of prematurity and neurodevelopmental outcome and quality of life at 10 years of age

**DOI:** 10.21203/rs.3.rs-4324566/v1

**Published:** 2024-05-08

**Authors:** Sudhir Sriram, Elizabeth Jensen, Michael Msall, Joe Yi, Vasyl Zhabotynsky, Robert Joseph, Karl Kuban, Jean Frazier, Stephen Hooper, Hudson Santos, Semsa Gogcu, Jeffrey Shenberger, Rebecca Fry, Thomas O’Shea

**Affiliations:** University of Chicago Hospitals; University of Chicago; University of North Carolina School of Medicine; North Carolina Comprehensive Cancer Care Center; Boston University Medical Center; Department Health Sciences School of Medicine; School of Nursing and health Studies; Wake Forest; Connecticut Children’s Hospital; UNC; University of North Carolina at Chapel Hill

## Abstract

**Objective:**

In a cohort of 10-year-old children born extremely preterm, we evaluated the hypothesis that increasing severity of retinopathy of prematurity (ROP) is associated with increasing frequency of unfavorable neurodevelopmental and quality of life outcomes.

**Study Design:**

Study participants were classified according to the severity of ROP. At 10 years of age, their neurocognitive abilities, academic achievement, and gross motor function were assessed, and they were evaluated for autism spectrum disorder, anxiety, depression, and quality of life.

**Results:**

After adjustment for sample attrition and confounders, only the association with lower quality of life persisted. Increasing severity of visual impairment was associated with worse neurodevelopmental outcomes and lower quality of life.

**Conclusion:**

Among extremely preterm children, severity of visual impairment, but not severity of ROP, was associated with adverse neurodevelopmental outcomes at 10 years of age. Both severe ROP and more severe visual impairment were associated with lower quality of life.

## Article Summary

Among children born extremely preterm, increased severity of retinopathy of prematurity was not associated with less optimal neurodevelopmental outcomes but was associated with less optimal quality of life.

## Introduction

Survival of extremely low gestational age infants (ELGANS < 28 weeks gestational age) has improved over the past three decades.^1–4^ Survivors are at high risk for retinopathy of prematurity (ROP), with a prevalence of 10–33%.^2,5^ In most cases, ROP resolves without serious visual sequelae, however, severe ROP is associated with worse cognitive and motor abilities in early childhood,^5–7^ even after adjusting for neonatal brain injury. Further, since the brain and eye share embryonic origins, risk factors associated to ROP such as oxidative stress and inflammation might have harmful effects on the development of the central nervous system.^8,9^ Nonetheless, few studies of neurodevelopmental outcomes among children with a history of ROP have followed individuals into older childhood.^10,11^

The follow up study of Cryotherapy for Retinopathy of Prematurity (CRYO-ROP) demonstrated that very low birth weight infants who develop threshold ROP are at increased risk of unfavorable visual outcomes and functional limitations in self-care, continency, mobility, communication, and social cognition skills at five years of age.^6^ At 8 years of age, CRYO-ROP study participants with unfavorable visual status had worse developmental, educational, and social skills than those who had favorable visual status.^12^ Furthermore, in a cohort of extremely low birth weight infants born in Victoria, Australia 1991–1992 (n = 180), study participants who previously had severe ROP exhibited more impairments at 17–18 years of age than peers without severe ROP on measures of visual processing, visual-motor integration, visual learning, IQ, and academic achievement tests in reading, spelling, and math; after controlling for perinatal risk factors, only visual acuity scores remained significant between the groups^10^ These studies describing the relationship between severe ROP and neurodevelopmental outcomes in school-aged children did not include children born within the past two decades. Recently, a secondary analysis of data collected from the South Korean National Health Insurance Service (KNHIS) data base throughout the 10-year follow-up found a higher prevalence of developmental delay in children with severe ROP compared with those mild ROP, although this study was based on registry data unadjusted for neonatal morbidities (mechanical ventilation, necrotizing enterocolitis, bronchopulmonary dysplasia, periventricular leukomalacia, and severe intraventricular hemorrhage) which may contribute to neurodevelopmental impairments.^11^

To increase the understanding of associations between severe ROP and neurocognitive, neurobehavioral and quality of life outcomes at school age, we analyzed the data from the ELGAN Study of neonates born before 28 weeks of gestation and prospectively followed to 10 years of age. We hypothesized that extremely preterm infants with severe ROP would have less favorable neurocognitive, neurobehavioral, and quality of life outcomes in middle childhood as compared to ELGAN participants without severe ROP. P

## Methods

### Participants

The ELGAN study is a multicenter, prospective, observational study of the risk of structural and functional neurologic disorders in extremely preterm infants.^13,14^ A total of 1506 infants born before the 28th week of gestation were enrolled during the years 2002–2004; 1222 were discharged alive from neonatal intensive care units (NICUs) and 1198 survived to 10 years. A subset of survivors from whom data were collected on neonatal systemic inflammation (n = 966; 81% of survivors) was recruited for evaluation at 10 years of age. Of those recruited, 889 (92%) returned for follow-up. After excluding 14 children who did not have retinal examination data to classify their level of ROP, our final sample was 875 children (73% of surviving members of the cohort).

### Retinopathy of Prematurity

Retinal examinations were performed by ophthalmologists experienced in ROP screening at each NICU where participants received care. Participating ophthalmologists helped to prepare a manual and data collection form, and then participated in efforts to minimize observer variability. Definitions of terms were those accepted by the International Committee for Classification of Retinopathy of Prematurity and the Committee for the Classification of Retinopathy of Prematurity (1984).^15–17^ In keeping with these guidelines, the first ophthalmologic examination was performed between the 31st and 33rd post-menstrual week. Follow-up exams were performed as clinically indicated until normal vascularization began in zone III. For the current analyses, severe ROP was defined as ROP stage 3,4, 5 and was stratified into those not requiring and those requiring surgery; less-severe ROP was defined as ROP stage 1, 2 and not requiring surgery. ELGAN participants without ROP served as the comparison group.

### Ten-Year Follow-Up Visit

Assessments were selected to provide the most comprehensive information about cognitive functions and selected academic skills. While the child was tested, the parent or caregiver completed a general health questionnaire (medical diagnoses and receipts of medications), as well as questionnaires about functional communication, social-emotional and adaptive behaviors, and quality of life.

### Eye disorders at ten years

At the time of the ten-year follow-up visit, a parent or guardian reported whether or not the child had these eye disorders: requirement for corrective lenses, blindness in one eye, blindness in both eyes, strabismus not requiring surgery, or strabismus requiring surgery. Based on these data, study participants are classified according to the most severe vision or eye problem using the following hierarchy: none, corrective lenses, strabismus not requiring surgery, strabismus requiring surgery, blind in one eye, or blind in both eyes.

### Cognitive function

To evaluate cognitive functioning at 10 years of age, we used the school-age Differential Ability Scales-II (DAS-II)^18^ and the “Developmental NEuroPSYchological Assessment” (NEPSY-II).^19^ Participants who were not able to obtain basal score on any given test because of severe motor impairment, were assigned the lowest score for the given test. We employed latent profile analysis to identify subgroups of extremely preterm (EP) children with similar profiles on 9 measures of verbal and nonverbal IQ (DAS-II Verbal and Nonverbal Reasoning scales), working memory (DAS-II Recall of Digits Backwards, Recall Sequential Order), concept generation and mental flexibility (NEPSY-II Animal Sorting), auditory attention and set switching (NEPSY-II Auditory Attention, Response Set), and simple inhibition and inhibition shifting (NEPSY-II Inhibition and Inhibition Switching). Using this approach, four neurocognitive profiles were identified : 1) “normal” - mean IQ and executive function scores within the normal range (< 0.5SD) on all measures; 2) “low-normal”-mean IQ and executive function scores ranged from 0.5–1 standard deviations below the mean in the normative sample; 3) “moderately impaired” - mean IQ and executive function scores between 1.5 and 2.5 standard deviations below the mean in the normative sample; and 4) “severely impaired” - mean IQ and executive function scores between 3 to 4 standard deviations below the mean in the normative sample.^20^ Academic achievement was evaluated using the Wechsler Individual Achievement Test-III (WIAT-III) Word Reading and Numerical Operations subtests.^21^

### Gross motor function

Gross motor function was assessed with Gross Motor Function Classification System (GMFCS), an ordinal scale that ranges from I to V, with higher levels indicative of greater functional limitations.^22^

### Epilepsy

Epilepsy was identified using a two-stage screening process that included administration of brief screening questionnaire to the parents, followed by an open-ended interview conducted by an epileptologist.^23,24^ A diagnosis of epilepsy required the consensus of 2 epileptologists and was defined as two or more seizures after the neonatal period that were not provoked by fever, trauma, or infection of the central nervous system.

### Autism assessment and Social Responsiveness Scales

Parents completed the Social Communication Questionnaire (SCQ) and if the score was greater than 11, they were asked to complete the Autism Diagnostic Interview, Revised (ADIR). Children who met the ADIR criteria for autism spectrum disorder (ASD) were evaluated with the Autism Diagnostic Observation Schedule 2 (ADOS2). All children who met standardized criteria for ASD on both ADIR and ADOS2 were classified as having ASD. ^25^ We used the Social Responsive Scale (SRS), completed by the parent, to assess a constellation of social challenges in social awareness, social cognition, social communication, social motivation and autistic mannerisms (stereotypies and restricted interests), behaviors observed in children with autism (35). As previously described, SRS was modeled as a binary outcome (≤ 65 or > 65).^26^ Scores > 65 indicate more impaired social responsiveness.

### ADHD

The parent or caregiver completed the Child Symptom Inventory (CSI-4) Parent Check List,^27^ and the child’s current teacher completed the teacher CSI-4 checklist. In order to parallel clinical decision making, three contexts in which ADHD symptoms could manifest were considered. Two of the contexts were taken from the CSI-4 norm-based cutoffs for the parent and teacher reports of ADHD symptoms, and the third context was based on the parent’s indication at interview of the child being diagnosed previously to have ADHD by a clinician. Participants were included in the ADHD symptom group if they met any 2 or 3 criteria.^28,29^

### Anxiety and Depression

For identification of anxiety disorders and depressive disorders, parents completed the CSI-4 parent check list and teachers completed CSI-4 teachers check list.^27^ The forms asked for details about generalized anxiety, social phobia, depressive disorder or dysthymic disorder. For this analysis, we defined parent-reported anxiety as an average T-score > 60 across three CSI-4 scales: general anxiety, separation anxiety, social phobia, and parent-reported depression as an average T score > 60 across two CSI-4 scales: dysthymia and major depression. Teacher-reported anxiety and depression were defined analogously except that only general and anxiety and social phobia were included in the definition of anxiety, because the teacher-reported CSI-4 scale does not include items on which the T-score for separation anxiety is based.

### Pediatric Quality of Life Inventory

For the quality of life (QoL) measure, a parent or a caregiver completed the PedsQL 4.0 generic core scales at 10 year-follow-up.^30,31^ The 23-item PedsQL was designed to measure core dimensions of QoL in areas of physical (8 items), emotional (5 items), social (5 items) and school (5 items) with each item scored on a five-point Likert scale. Each dimension was summed and linearly transformed to a 0–100 scale with higher scales indicating better QoL. In the analyses reported here we dichotomized the total PedsQL using a cutoff of < 70, which is two standard deviations below the mean in the reference sample.

### Data Analysis

To analyze associations between severity of ROP and outcomes at 10 years of age we used chi square tests for univariate analysis, and logistic regression models for multivariable analyses. The strength of association was estimated as odds ratios and adjusted odds ratios (aOR) and 95% confidence intervals (CI), with individuals without ROP as the referent group.

We created directed acyclic graphs^32^ to identify the minimally sufficient adjustment sets of variables to include in multivariable regression models to close non-causal, biasing pathways (Figure S1). Multivariable analyses adjusted for maternal age, eligibility for government-subsidized health insurance, number of years of formal education completed by the mother at the time of the study participant’s birth, marital status (married versus unmarried), duration of mechanical ventilation, gestational age, sepsis, sex, and surgical necrotizing enterocolitis. In a sensitivity analysis we adjusted for the set of variables just listed plus maternal fever during pregnancy. We expressed results as odds ratios with 95% confidence intervals. In sensitivity analyses we applied parametric multiple imputation to evaluate the potential for bias resulting from informative missingness due to sample attrition. Missing values were imputed using the adjustment covariates, and ten iterations of imputed datasets were analyzed; final odds ratios and p-values represent the mean odds ratios and p-values of the ten iterations. Statistical significance was defined as p < 0.05.

## Results

### Study Participants

Children who were evaluated at 10 years of age (n = 889), as compared to members of the ELGAN cohort who survived to 10 years of age (1198) were similar in maternal variables except that the study sample had slightly lower proportions of participants born to mothers with high school education or less, Medicaid eligibility, and Black race. Infant variables were similar for those evaluated at ten years as compared to all surviving cohort members (Table S1). Of the 875 children included in the present analysis, 16% (n = 138) had severe ROP (stages 3, 4, 5) requiring surgery; 15% (n = 129) had severe ROP not requiring surgery; 44% (n = 385) had less severe ROP (stages 1, 2) not requiring surgery; and 25% (n = 223) did not have ROP (Table 1). Severity of ROP was associated with having an unmarried mother, Hispanic ethnicity, lower gestational age and birth weight, sepsis, necrotizing enterocolitis, and chronic lung disease (p < 0.05) (Table 1).

### ROP Severity and Outcomes at 10 years of age

Table 2 summarizes the frequencies of eye or vision problems and frequencies of cognitive, behavioral, neurological, and quality of life outcomes as a function of severity of ROP. In general, as compared to individuals without ROP, those with less severe ROP had similar frequencies of eye problems; those who required surgery for ROP had the highest frequencies of problems; and those with more severe ROP who did not require surgery had frequencies intermediate between those with no ROP and those who required surgery.

Table 3 presents unadjusted and adjusted odds ratios for associations between ROP severity and adverse outcomes at 10 years. As compared to no ROP, less severe forms of ROP were not associated with outcomes at 10 years. In contrast, more severe ROP requiring surgery was associated with lower scores for numeric operations, higher likelihood of parent-reported anxiety, and lower scores for quality of life. More severe ROP that did not require surgery was associated with lower scores for quality of life but not with adverse neurodevelopmental outcomes. When maternal fever was added as a covariate in the sensitivity analysis (Table S2), the results remained the same.

### Relationship of eye disorders/vision problems with outcomes at 10 years

Table 4 highlights the associations between any vision or eye problem and outcomes at 10 years. Study participants are classified according to the most severe vision or eye problem using the following hierarchy: none, corrective lenses, strabismus not requiring surgery, strabismus requiring surgery, blind in one eye, or blind in both eyes.

Overall, we found that the frequencies of adverse cognitive, behavioral, and neurological outcomes were similar in groups with current eye or vision problems as compared to groups without current eye or vision problems, unless the individual was blind in one or both eyes (Table 4).

Table 5 presents unadjusted odds ratios for associations between eye problems and adverse outcomes at 10 years. Children who required corrective lenses but did not have strabismus or blindness (25.4% of participants), were more likely to have cognitive impairment and had lower scores for quality of life. Children who had strabismus but did not require surgery and were not blind (7.8% of participants), had higher odds of autism spectrum disorder. Children who had strabismus and required surgery (6.5% of participants) for strabismus were more likely to have lower scores for numeric operations, epilepsy, and lower scores for quality of life. Lastly, children with blindness in one or both eyes (7% of participants) were more likely to have cognitive impairment, low academic achievement test scores for numeric operations and word reading, low social responsiveness, epilepsy, and low quality of life.

## Discussion

In this cohort of children born before 28 weeks of gestation, we found that the severity of visual impairment, was associated with the risk of adverse neurodevelopmental outcomes and with quality of life at 10 years of age. In contrast, severity of ROP during the neonatal period was minimally predictive of cognitive, academic, or behavioral outcomes once controlling for confounding medical and social variables. On the other hand, severity of ROP was associated with overall quality of life as reported by the parent. Thus, while associations were not found between ROP severity and outcomes evaluated with objective assessments associations were found for the more subjective outcome of quality of life, an important outcome from the perspective of the individual and family.^33^

Among participants in the large CRYO-ROP study, poor vision after threshold ROP was associated with an increased risk for disability^6^ as well as worse educational outcomes and social skills at age 8 years.^12^ At age 10 years, parental perspectives on health status and health-related quality of life were described in 244 participants with severe (i.e., threshold) ROP and 102 participants who did not develop ROP. Children with severe ROP were more likely to have functional limitations in vision, hearing, speech, ambulation, dexterity, emotion, cognition, and pain and reduction in health-related quality of life (HRQL). In agreement with our findings, among children who developed threshold ROP, those with poor visual outcomes had lower HRQL scores than those with better visual outcomes.^34^

Several other studies report an association between severe ROP and neurodevelopmental disabilities in children^35–38^ including cognitive impairment and delayed psychomotor development.^7,39–41^ In contrast, and consistent with our analyses, Altendahl et al. found no association between severe ROP and neurodevelopmental outcome at 12, 24 and 36 months of age, after adjusting for birth weight, male sex, intraventricular hemorrhage, and eligibility for public health insurance.^42^ In another recent study of extremely preterm children, Brumbaugh et al. found that those with ROP stage ≤ 3 that regressed without intervention were no more likely to have adverse neurodevelopmental at 2 years corrected age than children born extremely preterm without a history of ROP. By contrast, children who underwent an ophthalmologic intervention for ROP had worse neurodevelopmental outcomes at 2 years corrected age than did children born extremely preterm who did not undergo intervention for ROP.^43^ Sources of variation across studies of the relationship of developmental outcomes to ROP include differences in years and locations of births, neonatal treatments and co-morbidities, outcome assessments, and control of confounding. In addition, educational services and neuroplasticity, manifesting as improvement in neurodevelopmental functions,^44^ may attenuate associations between ROP, related visual deficits, and neurodevelopmental outcomes in middle childhood.

Our study has several strengths, including its large multi-center sample, making it unlikely that we missed important associations because of lack of statistical power. Second, a broad range of outcomes, including neuromotor, neurocognitive, neurobehavioral, and quality of life, were collected prospectively by examiners who were unaware of study participant’s ROP status. Third, we selected infants based on gestational age, not birth weight, to minimize confounding due to factors related growth restriction. A limitation of our study is the sample attrition between the time of NICU discharge and follow-up. An additional limitation is the lack of information about visual acuity, which precluded our ability to investigate whether deficits in visual acuity mediate the association that we observed between ROP severity and quality of life in middle childhood.

In summary, ten years after birth, extremely low gestational age neonates who had severe ROP, as compared to neonates without this condition, were more likely to have low scores on overall pediatric quality of life. It is reassuring that in general, cognitive, neuromotor, and behavioral outcomes did not differ significantly as a function of ROP severity. Worse neurocognitive outcomes were associated with severe visual impairment (strabismus requiring surgery or blindness in one of both eyes). These findings could serve as the basis for cautious optimism when counselling families of extremely preterm infants who have experienced severe ROP without severe visual impairment dysfunction.

## Figures and Tables

**Table 1:** Maternal and neonatal characteristics as a function of severity of retinopathy of prematurity. All percentages are Column totals, based on denominators that include participants with missing values.

		Severity of Retinopathy of Prematurity				
Characteristics		No ROP (n = 223)	Less severe ROP; no surgery (n = 385)	More severe ROP no surgery (n = 129)	ROP requiring surgery (n = 138)	p value
Maternal age (years)	< 21	20 (9.0%)	58 (15.1%)	12 (9.3%)	22 (15.9%)	0.1
	21–35	154 (69.1%)	248 (64.4%)	96 (74.4%)	88 (63.8%)	
	> 35	49 (22.0%)	79 (20.5%)	21 (16.3%)	28 (20.3%)	
Unmarried mother		91 (40.8%)	161 (41.8%)	54 (41.9%)	40 (29.0%)	0.05
Maternal education (years)	≤ 12	78 (35.9%)	165 (44.2%)	49 (39.2%)	58 (43.3%)	0.1
	> 12 to < 16	47 (21.7%)	82 (22.0%)	31 (24.8%)	39 (29.1%)	
	≥ 16	92 (42.4%)	126 (33.8%)	45 (36.0%)	37 (27.6%)	
Mother Covered by Medicaid or Another State-supported Medical Insurance		71 (32.3%)	142 (37.4%)	48 (38.1%)	40 (29.6%)	0.3
Race	Asian, Native American, or mixed race	22 (9.9%)	45 (11.8%)	15 (11.8%)	14 (10.4%)	0.6
	Black	56 (25.2%)	95 (24.9%)	41 (32.3%)	31 (23.1%)	
	White	144 (64.9%)	242 (63.4%)	71 (55.9%)	89 (66.4%)	
Hispanic	No	209 (93.7%)	348 (90.9%)	112 (87.5%)	118 (85.5%)	0.05
	Yes	14 (6.3%)	35 (9.1%)	16 (12.5%)	20 (14.5%)	
Mother’s Pre-pregnancy Body Mass Index (kg/m^2^)	< 18.5	19 (8.7%)	33 (8.9%)	12 (9.8%)	4 (3.0%)	0.2
	18.5 to < 30	148 (67.6%)	262 (70.8%)	78 (63.9%)	98 (73.7%)	
	≥ 30	52 (23.7%)	75 (20.3%)	32 (26.2%)	31 (23.3%)	
clinical chorioamnionitis		111 (55.0%)	189 (52.5%)	56 (48.3%)	78 (60.0%)	0.3
Indication for delivery	Preterm labor	102 (45.7%)	182 (47.3%)	59 (45.7%)	62 (44.9%)	0.2
	Premature rupture of membranes	59 (26.5%)	77 (20.0%)	25 (19.4%)	26 (18.8%)	
	Preeclampsia	25 (11.2%)	44 (11.4%)	27 (20.9%)	16 (11.6%)	
	Placental abruption	19 (8.5%)	46 (11.9%)	11 (8.5%)	15 (10.9%)	
	Cervical insufficiency	12 (5.4%)	18 (4.7%)	4 (3.1%)	10 (7.2%)	
	Fetal indication	6 (2.7%)	18 (4.7%)	3 (2.3%)	9 (6.5%)	
Cesarean delivery		143 (64.1%)	259 (67.3%)	88 (68.2%)	91 (65.9%)	0.8
Sex	Female	103 (46.2%)	192 (49.9%)	60 (46.5%)	71 (51.4%)	0.7
	Male	120 (53.8%)	193 (50.1%)	69 (53.5%)	67 (48.6%)	
Multiple gestation		79 (38.3%)	127 (34.8%)	42 (35.0%)	43 (32.3%)	0.7
Gestational age (weeks)	23–24	10 (4.5%)	74 (19.2%)	35 (27.1%)	68 (49.3%)	< .0001
	25–26	90 (40.4%)	178 (46.2%)	71 (55.0%)	58 (42.0%)	
	27	123 (55.2%)	133 (34.5%)	23 (17.8%)	12 (8.7%)	
Birth weight (grams)	≤ 750	33 (14.8%)	138 (35.8%)	69 (53.5%)	91 (65.9%)	< 0.001
	750–1000	104 (46.6%)	175 (45.5%)	53 (41.1%)	42 (30.4%)	
	> 1000	86 (38.6%)	72 (18.7%)	7 (5.4%)	5 (3.6%)	
Birth weight z-score	< −2	6 (2.7%)	19 (4.9%)	13 (10.1%)	15 (10.9%)	
	≥ −2, < −1	13 (5.8%)	57 (14.8%)	25 (19.4%)	24 (17.4%)	< 0.001
	>= −1	204 (91.5%)	309 (80.3%)	91 (70.5%)	99 (71.7%)	
White matter injury on neonatal ultrasound†		45 (20.2%)	74 (19.2%)	29 (22.5%)	38 (27.5%)	0.2
Definite Sepsis		57 (25.6%)	102 (26.5%)	50 (38.8%)	48 (34.8%)	0.0003
Presumed Sepsis		59 (26.5%)	136 (35.3%)	48 (37.2%)	46 (33.3%)	
No sepsis		107 (48.0%)	147 (38.2%)	31 (24.0%)	44 (31.9%)	
No NEC		193 (86.5%)	305 (79.2%)	86 (66.7%)	92 (66.7%)	< 0.001
Necrotizing enterocolitis, medical		24 (10.8%)	57 (14.8%)	29 (22.5%)	27 (19.6%)	
Necrotizing enterocolitis, surgical		5 (2.2%)	11 (2.9%)	5 (3.9%)	12 (8.7%)	
Spontaneous intestinal perforation		1 (0.4%)	12 (3.1%)	9 (7.0%)	7 (5.1%)	
Chronic lung disease¶		60 (27.1%)	194 (50.5%)	91 (70.5%)	114 (82.6%)	< 0.001
SNAP score	< 20	145 (66.5%)	206 (54.6%)	54 (42.2%)	44 (32.1%)	< 0.001
	20–29	45 (20.6%)	85 (22.5%)	38 (29.7%)	44 (32.1%)	
	30+	28 (12.8%)	86 (22.8%)	36 (28.1%)	49 (35.8%)	

† white matter injury was defined as one or more of the following findings, with agreement by two readers, on cranial ultrasound: persistent ventricular enlargement beyond the first month of life, echodensity in the periventricular white matter, echolucency in the periventricular white matter

¶ chronic lung disease was defined as receipt of supplemental oxygen at 36 weeks post-menstrual age

Abbreviations: NICU – neonatal intensive care unit; IQ – intelligence quotient; NICU-neonatal intensive care unit; SIP – spontaneous intestinal perforation; PMA – postmenstrual age; SNAP – Score for Neonatal Acute Physiology

**Table 2:** Outcomes at 10 years of age as a function of ROP severity.

Outcomes	Severity of Retinopathy of Prematurity			
	No ROP (n = 223)	Less severe ROP; no surgery (n = 385)	More severe ROP no surgery (n = 129)	ROP requiring surgery (n = 138)
Any eye or vision problem at 10 years of age	149 (66.8%)	242 (62.9%)	58 (45.0%)	15 (10.9%)
Eyeglasses	50 (22.4%)	89 (23.1%)	33 (25.6%)	52 (37.7%)
Strabismus	13 (5.8%)	25 (6.5%)	12 (9.3%)	18 (13.0%)
Strabismus surgery	7 (3.1%)	21 (5.5%)	11 (8.5%)	18 (13.0%)
Blind in one eye	0 (0.0%)	6 (1.6%)	9 (7.0%)	13 (9.4%)
Blind in both eyes	4 (1.8%)	2 (0.5%)	6 (4.7%)	22 (15.9%)
Normal cognitive function	91 (41.2%)	132 (34.8%)	38 (29.7%)	33 (24.8%)
Low Normal cognitive function	90 (40.7%)	157 (41.4%)	56 (43.8%)	51 (38.3%)
Moderately Impaired cognitive function	32 (14.5%)	68 (17.9%)	19 (14.8%)	26 (19.5%)
Severely Impaired cognitive function	8 (3.6%)	22 (5.8%)	15 (11.7%)	23 (17.3%)
Word Reading < 70	18 (8.3%)	33 (8.9%)	19 (15.2%)	26 (20.0%)
Numerical Operations < 70	22 (10.0%)	54 (14.3%)	24 (19.0%)	34 (26.0%)
Autism spectrum disorder	7 (3.2%)	26 (6.8%)	14 (11.4%)	13 (10.7%)
SRS ≥ 65 (children without ASD and with IQ ≥ 70)	22 (9.9%)	32 (8.3%)	16 (12.4%)	16 (11.6%)
ADHD	191 (86.4%)	307 (80.4%)	105 (83.3%)	106 (80.9%)
Anxiety-parent report	12 (5.4%)	44 (11.5%)	11 (8.8%)	23 (17.7%)
Depression-parent report	6 (2.7%)	17 (4.5%)	5 (4.0%)	7 (5.4%)
Anxiety-teacher report	10 (6.3%)	27 (9.5%)	7 (8.0%)	6 (6.0%)
Depression-teacher report	9 (5.7%)	12 (4.3%)	3 (3.4%)	0 (0.0%)
Epilepsy	19 (8.5%)	23 (6.0%)	7 (5.4%)	17 (12.4%)
GMFCS ≥ 3	8 (3.6%)	12 (3.1%)	6 (4.7%)	18 (13.0%)
Peds quality of life < 70	38 (17.3%)	84 (22.0%)	43 (34.1%)	47 (35.6%)

Abbreviations: ROP – retinopathy of prematurity; ADHD – attention deficit hyperactivity disorder; SRS – Social Responsiveness Scale (Scores ≥ 65 indicate more impaired social responsiveness); ASD – autism spectrum disorder; IQ – intelligence quotient; GMFCS – Gross Motor Function Classification System

**Table 3:** Associations between severity of retinopathy of prematurity and outcomes at 10 years of age. Referent group is infants without ROP (n = 223). Data are unadjusted odds ratios (95% confidence intervals in parentheses). Bold indicates p < 0.05.

	Unadjusted OR			Adjusted OR†		
	Less severe ROP no surgery Vs No ROP	More severe ROP no surgery Vs No ROP	ROP requiring surgery Vs No ROP	Less severe ROP no surgery Vs No ROP	More severe ROP no surgery Vs No ROP	ROP requiring surgery Vs No ROP
Cognitive impairment	1.41 (0.93, 2.14)	1.64 (0.97, 2.75)	**2.64 (1.61, 4.31)**	1.04 (0.65, 1.65)	0.94 (0.52, 1.71)	1.58 (0.88, 2.84)
Word Reading < 70	1.08 (0.59, 1.97)	**1.99 (1.00, 3.96)**	**2.78 (1.46, 5.30)**	0.72 (0.37, 1.39)	1.00 (0.46, 2.17)	1.54 (0.71, 3.33)
Numerical Operations < 70	1.50 (0.89, 2.54)	**2.12 (1.13, 3.96)**	**3.15 (1.75, 5.68)**	1.20 (0.68, 2.13)	1.42 (0.70, 2.89)	**2.28 (1.13, 4.59)**
Autism spectrum disorder	2.21 (0.94, 5.19)	**3.87 (1.52, 9.87)**	**3.59 (1.39, 9.27)**	1.48 (0.59, 3.69)	2.10 (0.75, 5.86)	1.69 (0.58, 4.95)
SRS ≥ 65 (children without ASD and with IQ ≥ 70)	0.83 (0.47, 1.46)	1.29 (0.65, 2.56)	1.20 (0.61, 2.37)	0.80 (0.44, 1.46)	1.37 (0.64, 2.95)	1.54 (0.68, 3.46)
ADHD	1.55 (0.98, 2.46)	1.27 (0.69, 2.33)	1.50 (0.84, 2.68)	1.25 (0.76, 2.05)	0.85 (0.44, 1.66)	1.01 (0.51, 2.00)
Anxiety-parent report	**2.27 (1.17, 4.40)**	1.68 (0.72, 3.93)	**3.74 (1.79, 7.81)**	**2.26 (1.13, 4.52)**	1.57 (0.63, 3.93)	**4.08 (1.74, 9.55)**
Depression-parent report	1.67 (0.65, 4.31)	1.49 (0.45, 5.00)	2.04 (0.67, 6.20)	1.59 (0.59, 4.28)	1.42 (0.38, 5.25)	1.96 (0.54, 7.11)
Anxiety-teacher report	1.58 (0.75, 3.36)	1.30 (0.48, 3.53)	0.96 (0.34, 2.72)	1.37 (0.62, 3.04)	1.07 (0.36, 3.16)	0.68 (0.21, 2.22)
Depression-teacher report	0.74 (0.30, 1.79)	0.58 (0.15, 2.22)	Low sample size	0.81 (0.30, 2.15)	0.69 (0.16, 2.98)	Low sample size
Epilepsy	0.68 (0.36, 1.28)	0.62 (0.25, 1.51)	1.52 (0.76, 3.04)	**0.49 (0.25, 0.98)**	**0.37 (0.14, 0.97)**	0.99 (0.43, 2.26)
GMFCS ≥ 3	0.86 (0.35, 2.15)	1.31 (0.44, 3.87)	**4.03 (1.70, 9.55)**	0.59 (0.22, 1.57)	0.70 (0.22, 2.27)	2.12 (0.76, 5.92)
Peds quality of life < 70	1.35 (0.89, 2.07)	**2.48 (1.49, 4.12)**	**2.65 (1.61, 4.36)**	1.11 (0.70, 1.76)	**1.79 (1.01, 3.16)**	**1.95 (1.08, 3.52)**

Abbreviations: ROP – retinopathy of prematurity; ADHD – attention deficit hyperactivity disorder; SRS – Social Responsiveness Scale, (scores ≥ 65 indicates more social challenges); ASD – autism spectrum disorder; IQ – intelligence quotient; GMFCS – Gross Motor Function Classification System

† Multivariable analyses adjusted for maternal age, eligibility for government-subsidized health insurance, maternal years of formal education, marital status (married versus unmarried), duration of mechanical ventilation, gestational age, any early or late sepsis, and surgical NEC(Penrose drain, Exploratory Laparotomy, or both)

**Table 4:** Associations between any vision/eye problem and outcomes at 10 years. Study participants are classified according to the most severe vision/eye problem based on the following hierarchy: none/corrective lenses/strabismus not requiring surgery/strabismus requiring surgery/blind in one eye/blind in both eyes. P values are for chi square test for difference in proportions within rows.

Developmental outcome at 10 years	No vision/eye problem N = 474	corrective lenses n = 226	Strabismus, no surgery N = 69	Strabismus, surgery N = 58	Blind, one eye N = 28	Blind, both eyes N = 34	P value
Cognitive impairment	94 (20.1%)	62 (27.7%)	7 (10.4%)	18 (31.0%)	12 (46.2%)	21 (67.7%)	< .0001
Low score Word reading	44 (9.6%)	21 (9.5%)	2 (3.1%)	7 (12.1%)	8 (30.8%)	15 (51.7%)	< .0001
Low score numerical operations	56 (12.0%)	38 (17.0%)	3 (4.5%)	15 (25.9%)	10 (38.5%)	13 (44.8%)	< .0001
Autism spectrum disorder	24 (5.2%)	13 (5.9%)	7 (10.3%)	5 (8.9%)	8 (30.8%)	4 (19.0%)	< .0001
SRS ≥ 65 (among those with no ASD and IQ ≥ 70)	37 (8.3%)	25 (11.4%)	12 (18.2%)	9 (16.7%)	1 (4.3%)	3 (15.0%)	0.07
ADHD	70 (15.0%)	44 (19.7%)	12 (17.6%)	13 (22.4%)	5 (18.5%)	8 (26.7%)	0.3
Anxiety-parent	42 (9.0%)	27 (12.1%)	8 (11.8%)	7 (12.1%)	1 (3.7%)	7 (23.3%)	0.1
Depression-parent	17 (3.7%)	10 (4.5%)	3 (4.4%)	3 (5.2%)	1 (3.7%)	1 (3.3%)	0.99
Anxiety-teacher	26 (7.8%)	15 (9.0%)	0 (0.0%)	4 (9.5%)	1 (4.5%)	4 (18.2%)	0.1
Depression-teacher	17 (5.1%)	4 (2.4%)	2 (3.8%)	0 (0.0%)	1 (4.5%)	1 (4.5%)	0.5
epilepsy	21 (4.4%)	16 (7.1%)	5 (7.2%)	8 (13.8%)	4 (14.3%)	12 (36.4%)	< .0001
GMFCS ≥ 3	15 (3.2%)	11 (4.9%)	2 (2.9%)	2 (3.4%)	2 (7.1%)	12 (35.3%)	< .0001
Peds Quality of Life < 70	78 (16.7%)	63 (28.3%)	18 (26.1%)	25 (43.1%)	10 (37.0%)	20 (66.7%)	< .0001

Abbreviations: SRS – Social Responsiveness Scale (higher scores indicate more social challenges); ASD – autism spectrum disorder; IQ – intelligence quotient; ADHD – attention deficit hyperactivity disorder; GMFCS – Gross Motor Function Classification System

**Table 5:** Odds ratios for associations between any vision/eye problem and outcomes at 10 years. Referent group is infants with no vision or eye problems (n = 474). Data are unadjusted odds ratios (95% confidence intervals in parentheses). Bold indicates p < 0.05.

Developmental outcome at 10 years	Eyeglasses only N = 226	Strabismus, no surgery N = 69	Strabismus, surgery N = 58	Blind, one eye N = 28	Blind, both eyes N = 34
Cognitive impairment	**1.52 (1.05, 2.20)**	0.46 (0.21, 1.05)	1.79 (0.98, 3.26)	**3.41 (1.53, 7.62)**	**8.35 (3.80, 18.3)**
Low score Word reading	0.99 (0.57, 1.71)	0.30 (0.07, 1.27)	1.29 (0.55, 3.03)	**4.19 (1.72, 10.2)**	**10.1 (4.58, 22.3)**
Low score numerical operations	1.50 (0.96, 2.35)	0.34 (0.10, 1.13)	**2.55 (1.33, 4.88)**	**4.56 (1.97, 10.6)**	**5.93 (2.71, 13.0)**
Autism spectrum disorder	1.47 (0.86, 2.51)	**2.49 (1.23, 5.04)**	2.17 (0.99, 4.76)	0.44 (0.06, 3.31)	1.14 (0.33, 3.92)
SRS > 65 (among those with no ASD and IQ ≥ 70)	1.14 (0.57, 2.28)	2.10 (0.87, 5.09)	1.80 (0.66, 4.92)	**8.15 (3.22, 20.6)**	**4.31 (1.35, 13.8)**
ADHD	1.40 (0.92, 2.12)	1.22 (0.62, 2.39)	1.64 (0.84, 3.20)	1.29 (0.47, 3.53)	2.07 (0.89, 4.83)
Anxiety-parent	1.39 (0.83, 2.32)	1.34 (0.60, 3.00)	1.38 (0.59, 3.24)	0.39 (0.05, 2.93)	**3.07 (1.24, 7.57)**
Depression-parent	1.24 (0.56, 2.75)	1.22 (0.35, 4.27)	1.44 (0.41, 5.06)	1.01 (0.13, 7.91)	0.91 (0.12, 7.07)
Anxiety-teacher	1.17 (0.60, 2.27)	0.00 (0.00, ****)	1.25 (0.41, 3.77)	0.56 (0.07, 4.36)	2.63 (0.83, 8.36)
Depression-teacher	0.46 (0.15, 1.39)	0.74 (0.17, 3.32)	0.00 (0.00, ****)	0.89 (0.11, 6.98)	0.89 (0.11, 6.98)
epilepsy	1.64 (0.84, 3.21)	1.69 (0.61, 4.63)	**3.45 (1.45, 8.20)**	**3.60 (1.14, 11.3)**	**12.3 (5.36, 28.4)**
GMFCS ≥ 3	1.57 (0.71, 3.47)	0.91 (0.20, 4.08)	1.09 (0.24, 4.90)	2.35 (0.51, 10.8)	**16.7 (6.98, 39.9)**
Peds Quality of Life < 70	**1.96 (1.34, 2.86)**	1.76 (0.97, 3.17)	**3.77 (2.12, 6.69)**	**2.93 (1.29, 6.63)**	**9.94 (4.48, 22.1)**

Abbreviations: SRS – Social Responsiveness Scale (higher scores indicate more social challenges); ASD – autism spectrum disorder; IQ – intelligence quotient; ADHD – attention deficit hyperactivity disorder; GMFCS – Gross Motor Function Classification System

## What’s known on this subject

Retinopathy of prematurity, a highly prevalent condition among extremely preterm neonates, has been associated with suboptimal neurodevelopmental outcomes, but studies of outcomes after infancy are limited.

## What this study adds:

This study suggests that after adjustment for confounding factors, increasing severity of retinopathy of prematurity is not associated with worse neurodevelopmental outcomes in middle childhood but is associated with lower quality of life.

## Abbreviations

ROP: retinopathy of prematurity
CRYO: ROP–Cryotherapy for Retinopathy of Prematurity
BMI: body mass index
CI: confidence interval
IQ: intelligence quotient
ADHD: attention deficit hyperactivity disorder
ELGAN: Extremely Low Gestational Age Newborn
CSI: 4–Child Symptom Index–4
NICU: neonatal intensive care unit
aOR: adjusted odds ratio
QoL: quality of life
